# Cancerous Communication between the Stomach and Colon

**Published:** 1845-04

**Authors:** 


### MEDICAL EXAMINER,

AND

### RECORD OF MEDICAL SCIENCE.

NEW SERIES—No. IV.—APRIL, 1845.

---

### ORIGINAL COMMUNICATIONS.

*Cancerous communication between the Stomach and Colon.* Extract of a letter from Dr. Wm. Waters, of Fredericktown, Maryland. (Communicated by Professor Dunglison.)

[Cases like the following are of deep interest to the physiologist and the pathologist. The communication which existed between the stomach and the colon, and the condition of the pylorus, must have interfered with the passage of aliment into the small intestine—the great seat of chylosis. Is the chyle, in such cases, formed and absorbed in the colon? Or may chylosis be effected, as suggested by Bouchardat and Sandras, and others, on certain aliments at least, in the stomach itself?]

V. D., aged 65 years, corpulent and of temperate habits. In July, 1843, he was attacked with symptoms of dyspepsia, such as flatulence, acid stomach, and some pain, heaviness, or sense of fulness across the epigastrium. These symptoms were frequently present until the 10th of January, 1844, when hæmatemesis occurred. It was evident the blood was of an arterial or vermilion colour. The discharge of blood was copious *sursum et deorsum.* At this time, the colic pains were pretty strong, which, with hemorrhage, were controlled by opium and acetate of lead in the first 24 hours. From this period the vomiting recurred occasionally, with irregular colicky pains, more especially about night, for several weeks. On one occasion the matter thrown up was observed by his wife to be puriform. (The hemorrhage and pus together led me to suspect organic disease in connection with his former dyspeptic symptoms for six months.) During this time his pulse was perfectly regular, but weak at times; occasionally cold extremities; no decided fever throughout his disease, but thirst in the very last stage, about ten days before dissolution. Tongue a little coated with a white fur almost through the whole progress of the case. The urgent symptoms were combated after reaction from hæmatemesis by cupping—wet and dry—sinapisms, and other counter-irritants, and occasionally a few doses of blue mass. This treatment was pursued during January and February, 1843, when the vomiting ceased, but occasionally he would feel pain, especially from improper diet. From this time, although his actual suffering was much abated and he was able to take exercise on foot, and in his carriage generally, yet the carcinomatous hue or lemon tint, evident in January last, was present throughout the whole course of his disease, attended with great emaciation, such as we usually see in tabes mesenterica. His full and corpulent person was reduced to a mere skeleton comparatively. In the case of Mr. D. there was less fever than in any organic disease of the stomach that I ever witnessed. He died on the 26th of December, 1844, 19 months after the first appreciable symptoms of dyspepsia.

*Autopsy, twenty-four hours after dissolution.* The tumour felt distinctly in this case a short time before death, between the ensiform cartilage and umbilicus, and more on the right than the left of the median line, was not near so distinct as during life. Omentum considerably absorbed and diseased. Stomach.—The pyloric end in a scirrhous state for more than two inches around and above the pylorus, and in some places more than an inch thick; the whole inner surface ulcerated; adherent to all adjacent parts, such as the liver for an inch square, and colon: here was a perforation of the stomach and tranverse colon about half an inch in diameter; the edges of the perforation scirrhous. Mesenteric glands indurated generally: other abdominal viscera healthy. Chest.—Lungs healthy; pericarditis had existed here, (as in every case of organic disease of the stomach I ever witnessed;) surface of the heart covered with coagulable lymph.

